# Synergistic Effect of Berberine-Based Chinese Medicine Assembled Nanostructures on Diarrhea-Predominant Irritable Bowel Syndrome *In Vivo*

**DOI:** 10.3389/fphar.2020.01210

**Published:** 2020-08-31

**Authors:** Lei Li, Herong Cui, Tong Li, Jinchai Qi, Hongshan Chen, Feng Gao, Xuehao Tian, Yunnong Mu, Rui He, Siyuan Lv, Fuhao Chu, Bing Xu, Penglong Wang, Haimin Lei, Hongri Xu, Chengxiang Wang

**Affiliations:** ^1^Respiratory Department, Beijing University of Chinese Medicine (BUCM) Third Affiliated Hospital, Beijing, China; ^2^School of Chinese Pharmacy, BUCM, Beijing, China; ^3^School of Acupuncture-Moxibustion and Tuina, BUCM, Beijing, China; ^4^School of Traditional Chinese Medicine, BUCM, Beijing, China; ^5^Emergency Department, BUCM Third Affiliated Hospital, Beijing, China

**Keywords:** berberine, self-assembly, nanostructures, diarrhea-predominant irritable bowel syndrome, microbiota–gut–brain axis

## Abstract

Diarrhea-predominant irritable bowel syndrome (IBS-D) is one common chronic functional disease of the digestive system with limited treatments. The microbiota–gut–brain axis (MGBA) has a central function in the pathogeny of IBS-D, which includes the participation of many various factors, such as brain-gut peptides (BGPs), immune inflammation, and intestinal flora. Inspired by the drug combination in traditional Chinese medicine (TCM), our previous study discovered that berberine (BBR) and baicalin (BA) could form natural self-assemblies as BA-BBR nanoparticles (BA-BBR NPs) and showed synergistic effects against IBS-D. Here, we investigated the synergistic effects of BA-BBR NPs on IBS-D model mice induced by chronic restraint stress plus Senna alexandrina Mill decoction with the influence on MGBA. BA-BBR NPs showed the best therapeutic effect on improving visceral hypersensitivity and diarrhea on IBS-D model mice, compared with BBR, BA, and BA/BBR mixture. Furthermore, BA-BBR NPs significantly (*P*<0.05) reduced the levels of 5-hydroxytryptamine (5-HT), vasoactive intestinal polypeptide (VIP) and choline acety transferase (CHAT) in colon tissues or of serum from BGPs; it lowered the expressions of the nuclear factor kappa-B (NF-κB) in colon tissues and changed the levels of basophil granulocyte (BASO) and leukomonocyte (LYMPH) in whole blood from immune inflammation; it altered the intestinal flora of Bacteroidia, Deferribacteres, Verrucomicrobia, Candidatus_Saccharibacteria, and Cyanobacteria from intestinal flora. In conclusion, BA-BBR NPs, after forming the natural self-assembly between BBR and BA, promoted the synergistic effect on IBS-D mice than the sum of BBR and BA effects, based to the formation of self-assemblies rather than the simple mixing. It further proved that synergistic effect of BA-BBR NPs on IBS-D mice might be related to BGPs, immune inflammation, and intestinal flora from three important interrelated components of MGBA. This study will provide a novel idea for the interpretation of TCM compatibility theory and provide the basis for BA-BBR NPs as a medicinal plant-derived natural and efficient nanomaterial for clinical use.

## Introduction

Irritable bowel syndrome (IBS) is an extremely common chronic non-organic disease of the digestive system (Chey et al., 2015). According to the Rome criteria IV, 5% to 7% of the general population suffers from IBS symptoms (Whitehead et al., 2017). Patients with IBS have greater risks of comorbidities, higher total medical expenditures, and lower health-related quality of life (Lee et al., 2016). IBS-D is the most common IBS subtype associated with recurrent abdominal pain and diarrhea (Drossman, 2016). MGBA disorder is an important pathological basis of IBS-D, which is the pathway of the nervous system to regulate the gastrointestinal tract and involved in the regulation of neural-endocrine-immune network systems, which is realized by BGPs. (Stasi et al., 2012; Oświęcimska et al., 2017). Studies suggest that this interaction seems to be influenced by multiple factors such as BGPs, immune inflammation, and intestinal flora (Tjong et al., 2011; Ringel and Maharshak, 2013). Although current treatment of IBS-D includes lifestyle and dietary interventions, antimotility drugs, probiotics, antispasmodics, and antidepressant medication (Ikechi et al., 2017). There is still no well-established treatment program for IBS-D that provided persistent relief for the multiple symptoms of IBS-D (Chey et al., 2015).

However, Chinese herbal therapies have been used to treat diarrhea for thousands years in eastern Asia (Chen et al., 2015). Generally, Chinese herbal medicine (CHM) including several herbals from traditional Chinese herbal formulae (CHF), which containing many different ingredients may act on multiple sites/pathways with potential synergistic effects and chemical reactions (Bi et al., 2017). Nowadays, it has been shown that CHM are effective in relieving symptoms among patients and rats with IBS-D (Mao et al., 2017; Fan et al., 2020). In particular, BBR alkaloid isolated from Coptis chinensis Franch rhizome, which has been widely used for the treatment of diarrhea, exhibit ameliorative effects on rats with IBS-D by modulating BGPs, inhibiting the intestinal inflammatory pathway and regulating the intestinal flora (Severina et al., 2001; Neag et al., 2018; Hou et al., 2019; Qiong et al., 2019). In addition, phytogenic BA, a dominant flavonoid isolated from the roots of Scutellaria *baicalensis* Georgi, is currently discussed as promising complementary agents in the prevention and treatment of intestinal and neurological diseases by neuroprotective, anti-inflammatory, and regulating intestinal flora (Ye et al., 2012; Zhang et al., 2018; Liang et al., 2019). Notably, the combination spirit of TCM has been widely accepted in clinic to reduce the side effect and improve efficacy (Song et al., 2013). In recent years, more and more researchers confirmed that combination of herbal medicines could enhance effects (Yue et al., 2017; Feng et al., 2018).

Inspired by the drug combination in TCM, our previous study discovered that BBR and BA could form natural self-assemblies in the form of precipitate (CFP) from Huang-Lian-Jie-Du-Tang (HLJDT). Moreover, we confirmed that BBR and BA could be self-assemble into nanoparticles in aqueous solution which induced by electrostatic interaction; and they were assembled to basic unit in a ratio of 1:1 (Wang et al., 2017). Natural and low-toxicity nanomaterial for clinical use has been a hot topic (Anastas and Eghbali, 2010). Unexpectedly, BA-BBR NPs showed the better effect on bacteriostatic activity significantly *in vitro*, comparing with BA/BBR mixture or BBR, respectively, and had good biocompatibility and safety (Li et al., 2019). In addition, it was noteworthy that BBR and BA were deduced from anti-diarrhea drug combination prescription of Scutellaria baicalensis Georgi − Coptis chinensis Franch rhizome combination, which has been used for thousands of years for intestinal disease in clinic in China (Chen et al., 2015). Hence, we hypothesized that BA-BBR NPs have synergistic effects on IBS-D, and our preliminary experiments have proved this.

Based on the effects of BBR, BA, and the TCM drug combination for synergistic effect on IBS-D, our previous work and nanomaterial encouraged us to further investigate the synergistic effect of a natural self-assembling nanoparticle formed by BBR and BA on IBS-D model mice with the influence on MGBA.

## Materials and Methods

### Drugs

Senna alexandrina Mill were purchased from Beijing Tcmages Pharmaceutical Co., Ltd. (Beijing, China), and identified by Professor Liu Chunsheng of Beijing University of Chinese Medicine. BBR (batch no. 110713-200208) and BA (batch no. 110715-200514) were all purchased from China Food and Drug Administration, (Beijing, China), with the quality score ≥ 98%.

### Animals

36 male SPF ICR mice, 8 to 12 weeks old, weighing 18 to 22 g, were purchased from Beijing Weitong Lihua Experimental Animal Technology Co., Ltd. (Animal Batch No: SCXK (Beijing) 2016-0006; Beijing, China). Standard mice food and tap water were available. During all experiments, mice were housed in groups of six animals. Water and food were available ad libitum. This study was conducted in accordance with the recommendations of the Guide for Care and Use of Laboratory Animals published by the U.S. National Research Council (National Research Council (US) Institute for Laboratory Animal Research, 1996), and Beijing University of Chinese Medicine Medical and Experimental Animal Ethics Committee. The protocol was approved by the Beijing University of Chinese Medicine Medical and Experimental Animal Ethics Committee. Animal ethics review number was BUCM-4-2019040401-2003.

### Reagents and Instruments

In this study, we used the following materials: sodium hydroxide, chloral hydrate, distilled water, normal saline, lubricant, formaldehyde, anhydrous ethanol, PBS buffer, 3% hydrogen peroxide solution, xylene were purchased from Bellen Chemistry Co., Ltd., (Beijing, China). hematoxylin dye solution, Servicebio G1004, hematoxylin separation solution, Servicebio G1039, hematoxylin blue return solution, ServicebioG1040, neutral gum, Servicebio G1403, staining kit DAB reagent Servicebio G1211, EDTA (PH8.0), antigen repair solution, Servicebio G1206, BSA, Servicebio G5001 were purchased from Nanjing Jingzhu Bio-technology Co., Ltd., (Nanjing, China). NFKB, Servicebio GB11142 1:200, HRP, Servicebio GB23303 1:200, ChAT checkerboard A079 were purchased from Nanjing Science and Technology Co., Ltd., (nanjing, China). mice serotonin (5-HT) elisa kit RGB - 60087M, VIP in mice elisa kit RGB - 60416M were purchased from Beijing Rui Lancet Bo Technology Development Co., LTD. (Beijing, China).

The following instruments were employed: constant temperature and humidity incubator (Cat. No: LHS-250HC-11; Shanghai Yiheng Scientific Instrument Co., Ltd., Shanghai, China), constant temperature oscillator (Cat. No: DDHZ-300; Suzhou Peiying Experimental Equipment Co., Ltd., Jiangsu, China), pressure steam sterilizer (Cat. No: MSG.N 180L; Shandong Weigao Group Medical Polymer Products Co., Ltd., Weihai, China), low speed centrifuge (Beijing Baiyang Medical Devices Co., Ltd., Beijing, China), precision electronic balance (Cat. No: BS124S; Beijing Sartorius Instrument System Co., Ltd., Beijing, China), and microplate reader (Cat. No: Multiskan MK3; Thermo Fisher Scientific Chemicals Co., Ltd.), plastic balloon (Beijing Tech Instrument Co., Ltd., Beijing, China), colorectal expansion pressure measuring device (Beijing Tech Instrument Co., Ltd., Beijing, China), dehydrator (JJ -12J, Junjie Electronics Co., Ltd., Wuhan, China), embedding machine (JB-P5, Junjie Electronics Co., Ltd., Wuhan, China), pathologic microtome (RM2016, Shanghai Leica Instrument Co., Ltd., Shanghai, China) Tissue spreading machine (KD-P, Shanghai Leica Instrument Co., Ltd., Shanghai, China), microwave oven galanz (P70D20TL-P4, Galanz Microwave Oven Electrical Appliances Co., Ltd., Shanghai, China), Automatic blood analyzer (Cat. No: XS-800i; SYSMEX CORPORATION Co., Ltd., Shanghai, China)

### Experimental Method

#### Preparation of Senna Decoction

Senna alexandrina Millwas weighed and boiled six times in water. After 10 min, the supernatant was obtained by filtering with double-layer gauze. After boiling at 100°C, the mass concentration was concentrated to 0.6 g/mL, stored in the refrigerator at −20°C, and heated in water bath at 25°C before drug administration every day.

#### Drug Preparation

BA-BBR NPs formation: BA sodium aqueous solutions was combined with BBR hydrochloride aqueous solution in the ratio of 1:1 (1 mM) at 80°C. We observed the instantaneous deepening of the color of the solution. At the moment of mixing, a large number of precipitates are precipitated out of the solution. We obtained the precipitate by centrifugation at 9000r, then washed three times with water and freeze dry. BA/BBR mixture formation: BA sodium aqueous solutions was combined with BBR hydrochloride aqueous solution in the ratio of 1:1 (1 mM).

#### IBS-D Model

After 1 week of acclimation, the IBS-D model was established by daily gavage of senna decoction (0.6 g/kg) plus restraint stress(for a duration of 1 h starting from 1 h after the gavage) for 2 weeks, as previously reported (Sun et al., 2015; Brun et al., 2017; Zhu et al., 2019). The experimental scheme was approved by the Laboratory Animal Ethics Committee of Beijing University of Chinese Medicine. After the model was established, the AWR score, the Bristol fecal character score, of which 2 points or more and 5 points or more, respectively, indicated the successful establishment of the IBS-D mice model.

#### Drug Intervention

**Figure 1:**
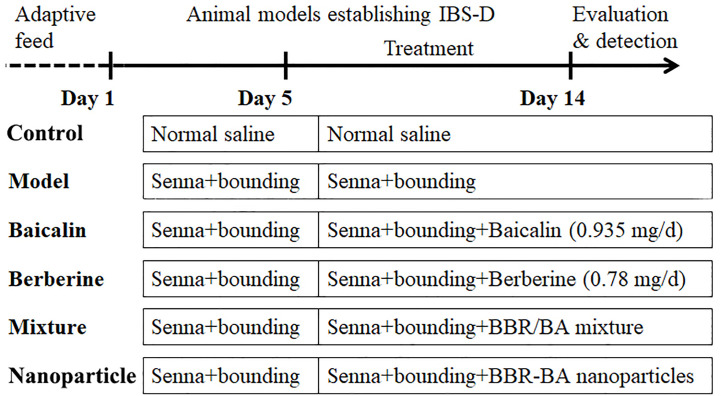
Diagram of administration.

Mice were randomly divided into six groups of six animals each as follows: Control group, Model group, BA group (0.935 mg/d), BBR group (0.78 mg/d) BA/BBR mixture group (1.715 mg/d) and BA-BBR NPs group (1.715 mg/d) delivered by gastric gavage for 10 d. The control and model groups were given the normal saline, 0.5 ml gavage, once a day for 10 d. The experimental process, the abbreviations, and descriptions of each group are shown in **Figure 1**.

#### Basic Efficacy Evaluation

The changes of general conditions of mice in the whole experiment were observed, including mental status, diet, stool, and hair. Then fecal traits were characterized using the Bristol stool typing score. Briefly, standard for the Bristol stool typing score was shown in **Table 1**. Finally, AWR score was measured as follows: Mice were anesthetized using 2% pentobarbital (0.04 ml/10 g) after being fasted for 12 h. The special dilated balloon was lubricated and carefully inserted into the mouse colon. Expanded the balloons slowly to constant pressure (15, 30, 45, and 60 mm Hg, with each pressure lasting 20 seconds, followed by a 4-minute stress-free interval) and recorded the results of the mice’s AWR score. The AWR scoring criteria was shown in **Table 2**.

**Table 1:** Fecal character Bristol classification and scoring criteria.

Grade	Fecal traits	Score
1	Dispersed similar to dry balls and nuts and was difficult to discharge	1
2	Sausage-shaped and multipiece	2
3	Sausage-like with a cracked surface	3
4	Sausage-like or serpentine, smooth, and soft	4
5	Soft clump with clear edges and was easy to discharge	5
6	A soft “sheet” with hairy edges or mushy consistency	6
7	Watery stool with no solid compounds	7

**Table 2:** AWR scoring criteria.

Abdominal retraction reflex	Score
The mice are emotionally stable and free to move	0
The mice are emotionally unstable and occasionally twist their heads	1
The abdominal muscles contracted slightly but the abdomen did not lift off the ground	2
The abdominal back muscles contracted strongly, and the abdomen was lifted off the ground	3
The abdominal back muscles contracted strongly, the back was arched, and the abdomen, pelvis and perineum were lifted off the ground	4

#### Histology

The hippocampal and colonic tissues of mice were collected on the 14th day, rinsed with normal saline, fixed with 10% formaldehyde, embedded in paraffin, and attached to the glass slide coated with poly-open amino acid. Paraffin sections of labeled mouse brain and colon tissues were routinely dewaxed to water, hematoxylin semen was stained, alcohol soluble eosin solution was added for staining, washed by running water, color separation, dehydration, transparency, sealing, dehydration with gradient alcohol, transparent xylene, neutral quick-drying adhesive sealing, and observed under light microscope.

#### NF-κB of Colonic Tissue by Immunohistochemistry (IHC)

Paraffin sections were dewaxed to water, antigens were repaired, endogenous peroxidase was blocked, serum was sealed, primary and secondary antibodies were added, color was obtained by diaminobenzidine (DAB), nuclei were re-dyed, dehydrated and sealed, microscopy was performed, and images were collected and analyzed. The nuclei stained with hematoxylin were blue, and the positive expression in DAB was brown-yellow.

#### CHAT of Serum by ELISA

Preparation of serum samples: the serum was centrifuged at 2500 RPM for 20 min. The prepared serum were taken, and the operation steps of the kit were strictly followed. Finally, the acetylcholine transferase content was calculated by using the formula.

#### 5-HT and VIP of Colon Tissue, Serum

Preparation of tissue samples to be measured: samples were homogenized sufficiently by homogenizer, 2500 RPM/min, and centrifuged for 20 min. Preparation of serum samples: the serum was centrifuged at 2500 RPM for 20 min. The prepared colonic tissue homogenate and serum were taken. The operation steps of the kit were strictly followed.

#### BASO and LYMPH of Whole Blood

Blood samples were collected from orbit of mice after anesthesia using 2% pentobarbital (0.04 ml/10 g) by anticoagulation tubes. The levels of BASO and LYMPH of whole blood were measured by Automatic blood analyzer.

#### 16S rRNA Gene Sequencing

Total bacterial genomic DNA samples were extracted following the manufacturer’s instructions. The quantity and quality of extracted DNA were measured using agarose gel electrophoresis, using Agencourt AMPure Beads (Beckman Coulter, Indianapolis, IN) and PicoGreen dsDNA Assay Kit (Invitrogen, Carlsbad, CA, USA). PCR ampliﬁcation of the bacterial 16S rRNA gene V4–V5 region as performed using the forward primer (5′-GTGYCAGCMGCCGCGGTAA-3′) and the reverse primer (5′-GGACTACHVGGGTWTCTAAT-3′). Sample-speciﬁc 7-bp barcodes were incorporated into the primers for multiplex sequencing. Amplicons were pair-end 2 × 300 bp sequencing using the Illlumina MiSeq platform with MiSeq Reagent Kit v3 at Shanghai Personal Biotechnology Co., Ltd (Shanghai, China). And the Quantitative Insights into Microbial Ecology (QIIME, v1.8.0) pipeline was employed to process the sequencing data. Raw sequencing reads with exact matches to the barcodes were assigned to samples and identiﬁed as valid sequences using the following ﬁltered criteria: sequences that had a length of <150 bp, sequences that had average Phred scores of <20, sequences that contained ambiguous bases and sequences that contained mononucleotide repeats of >8 bp. And paired-end reads were assembled using FLASH. The remaining high-quality sequences were clustered into operational taxonomic units (OTUs) at 97% sequence identity by UCLUST. Representative sequence was selected from each OTU using default parameters. Then, OTU taxonomic classiﬁcation was conducted by BLAST searching the representative sequences set against the Greengenes Database using the best hit. And, an OTU table was further generated to record the abundance of each OTU in each sample and the taxonomy of these OTUs. OTUs containing less than 0.001% of total sequences across all samples were then discarded. For minimizing the difference of sequencing depth across samples, an averaged, rounded rareﬁed OTU table was generated by averaging 100 evenly re-sampled OTU subsets under the 90% of the minimum sequencing depth for further analysis at Beijing HT Health Biotechnology Co., Ltd.

### Statistical Methods

One-way ANOVA or Kruskal Wallis Tests among groups was used for significant differences, and the data were expressed as mean ± Standard deviation. As well, Pearson’s correlation test was used to analyze the potential correlation. All the analyses were carried out with SPSS version 17.0 (SPSS 22.0, Chicago, IL, USA). Statistically significant differences were defined at *P* < 0.05.

## Results

### Basic Efficacy on IBS-D Mice

**Figure 2:**
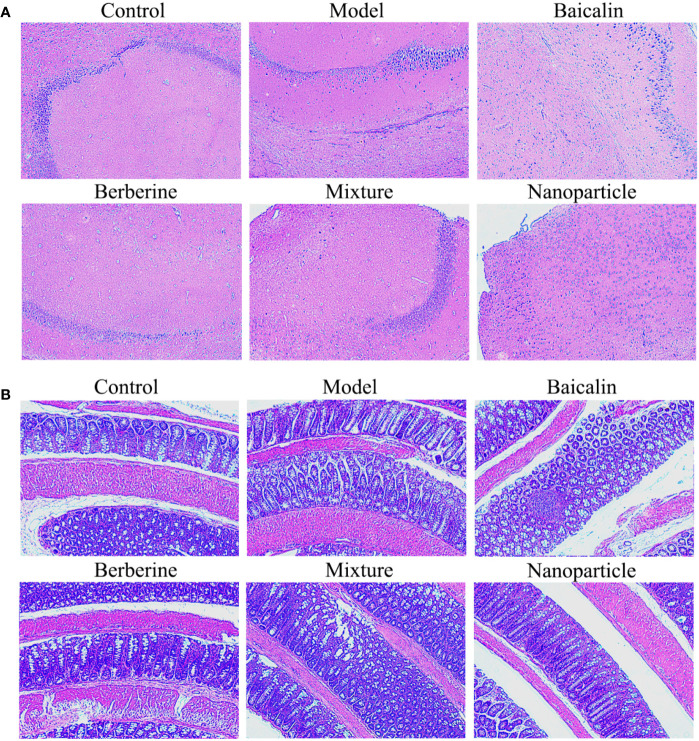
H&E of hippocampus and colon. **(A)** H&E of hippocampus. **(B)** H&E of colon.

General conditions were observed, and the Bristol fecal character score and AWR score were measured to assess the induction of IBS-D in mice. In addition, the colons of mice were stained with H&E to observe histological changes on hippocampus and colon of IBS-D mice in each group. The general conditions were not different between groups. The mice were sensitive, with good mental status, normal diet and stool, smooth and glossy hair in each group. After IBS-D induction, compared with control group, model group showed depression, easy stimulation, loss of appetite, varying degrees of loose stools and body hair contaminated by loose stool. After treatment, compared with model group, the above symptoms were alleviated in four treatment groups, and the BA-BBR NPs group was superior to the other groups. The Bristol fecal character score was not statistically different between groups (*P* > 0.05). After IBS-D induction, the Bristol fecal character score in model group was significantly increased than that in the control group (*P* < 0.05), and the comparison of the Bristol fecal character score in each group was statistically significant (*P* < 0.05). After drug treatment, the Bristol fecal character score were all decreased in treatment groups compared with model group (*P* < 0.05). Compared with other treatment groups, BA-BBR NPs group had the lowest Bristol score (*P* < 0.05) (**Table 3**). Moreover, there was no significant difference in AWR score between the groups before modeling (*P* > 0.05). After IBS-D induction, AWR score was increased in model group compared with control group, the comparison of AWR score in each group was statistically significant (*P* < 0.05). After drug treatment, AWR score were all decreased in treatment groups compared with model group (*P* < 0.05). Compared with other treatment groups, BA-BBR NPs group had the lowest AWR score (*P* < 0.05) (**Table 4**). Hematoxylin and eosin H&E staining showed that colonic epithelial cells and hippocampal cells in each group were arranged regularly. Moreover, no significant pathological changes were observed in any group (**Figure 2**).

**Table 3:** Evaluation table for Bristol fecal character score.

Group	Before modeling	After modeling	After treatment
Control	3.25 ± 0.67	3.55 ± 0.45	3.58 ± 0.62
Model	3.58 ± 0.65	6.40 ± 0.28*	6.35 ± 0.36*
Baicalin	3.55 ± 0.36	6.32 ± 0.45*	5.28 ± 0.39^#^
Berberine	3.64 ± 0.45	6.25 ± 0.54*	4.38 ± 0.36^#^
Mixture	3.25 ± 0.35	6.30 ± 0.25*	5.06 ± 0.28^#^
Nanoparticles	3.42 ± 0.58	6.35 ± 0.50*	3.82 ± 0.45^#^

ANOVA with the post hoc test was used to calculate the significance of the differences, * and ^#^ represents P < 0.05 compared with the control and model group, respectively. The results are expressed as the mean ± S.D.

**Table 4:** Evaluation table for AWR score.

Group	Before modeling	After modeling	After treatment
Control	1.65 ± 0.35	1.75 ± 0.24	1.58 ± 0.44
Model	1.58 ± 0.33	2.55 ± 0.21*	2.67 ± 0.31*
Baicalin	1.35 ± 0.39	2.95 ± 0.42*	2.09 ± 0.35^#^
Berberine	1.64 ± 0.41	2.63 ± 0.34*	2.08 ± 0.36^#^
Mixture	1.55 ± 0.32	2.83 ± 0.25*	2.16 ± 0.25^#^
Nanoparticles	1.72 ± 0.37	2.75 ± 0.52*	1.92 ± 0.32^#^

ANOVA with the post hoc test was used to calculate the significance of the differences, * and ^#^ represents P < 0.05 compared with the control and model group, respectively. The results are expressed as the mean ± S.D.

### Brain-Gut Peptides: 5-HT, VIP, CHAT of Colon Sections or Serum

**Figure 3:**
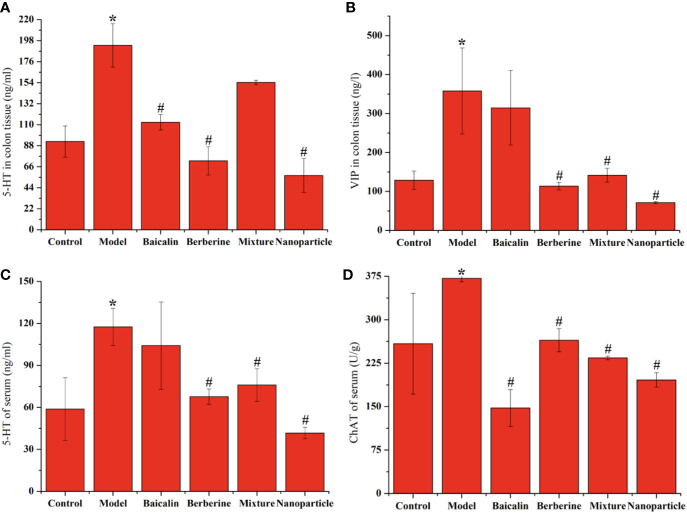
Levels of 5-HT, VIP or CHAT in colon tissue or serum **(A)** 5-HT of colonic tissue **(B)** VIP of colonic tissue **(C)** 5-HT of serum **(D)** CHAT of serum. ANOVA with the *post hoc* test was used to calculate the significance of the differences, * and ^#^ represents *P* < 0.05 compared with the control and model group, respectively. The results are expressed as the mean ± S.D.

Compared with the control group, the levels of 5-HT and VIP of colon sections in the model group increased significantly (*P* < 0.05). Compared with the model group, the levels of 5-HT and VIP of colon sections decreased in each administration group, with statistically significant differences (*P* < 0.05). Compared with the control group, the levels of 5-HT and CHAT of serum in the model group increased significantly (*P* < 0.05). Compared with the model group, the levels of 5-HT and CHAT of serum decreased in each administration group, with statistically significant differences (*P* < 0.05). Particularly, BA-BBR NPs is the superior efficacy by lowering the levels of 5-HT, VIP, and CHAT of serum or colon tissues (**Figure 3** and **Supplementary Materials Table S1-S4**).

### NF-κB of Colon Tissues by IHC and BASO and LYMPH in Whole Blood

**Figure 4:**
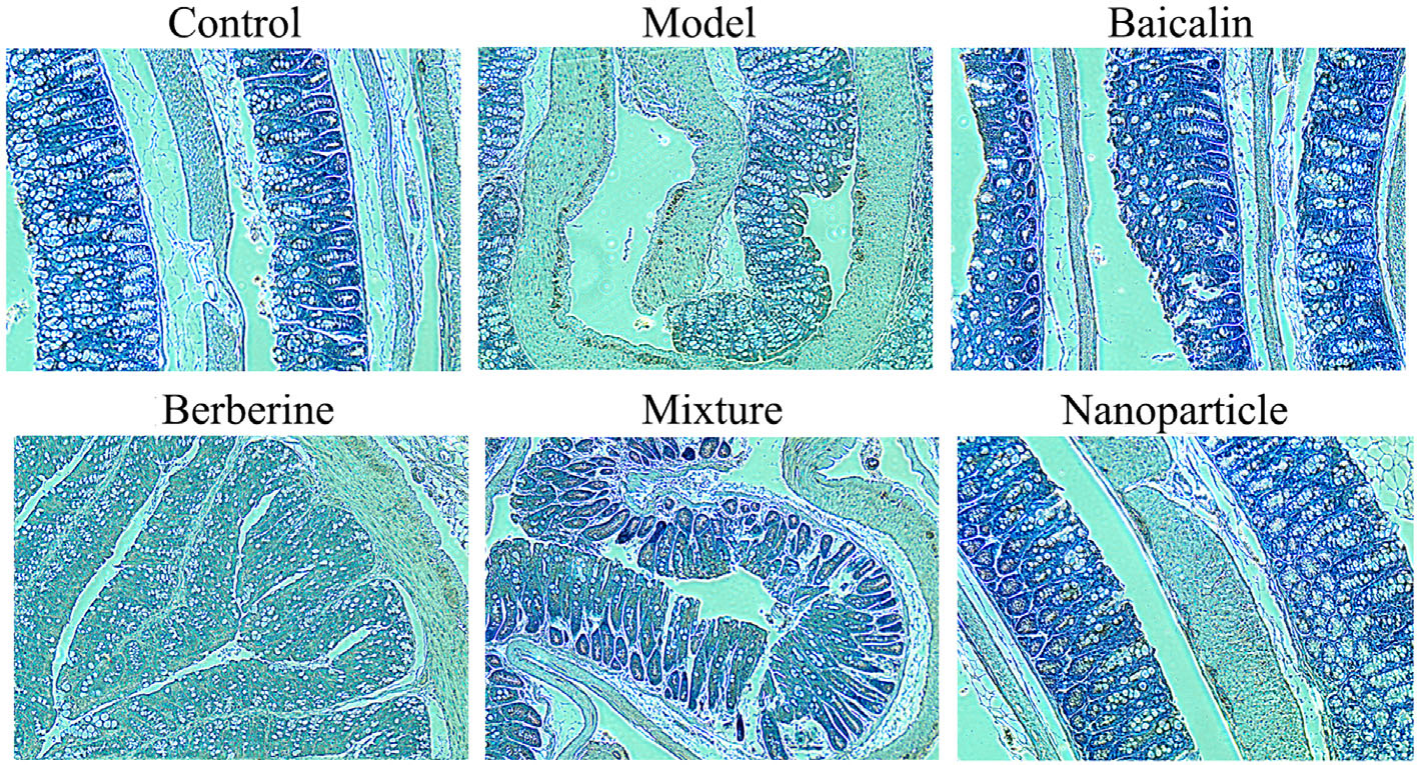
Expression of NF-κB in colon tissues.

**Figure 5:**
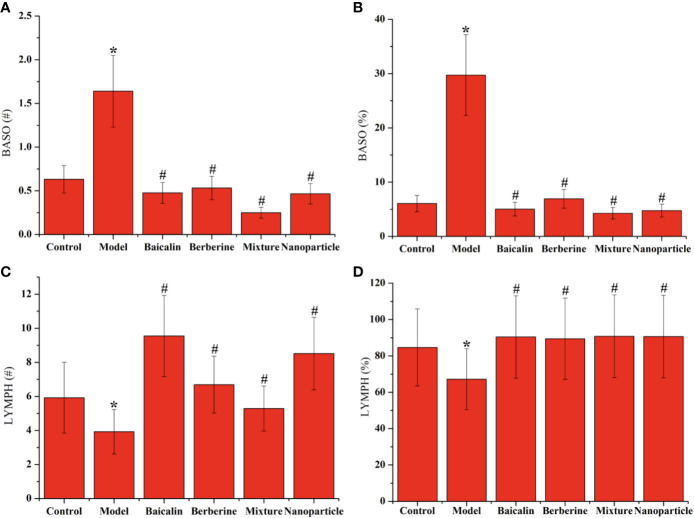
Levels of BASO and LYMPH in whole blood. **(A)** BASO# of whole blood. **(B)** BASO% of whole blood. **(C)** LYMPH# of whole blood. **(D)** LYMPH% of whole blood. ANOVA with the *post hoc* test was used to calculate the significance of the differences, * and ^#^ represents *P* < 0.05 compared with the control and model group, respectively. The results are expressed as the mean ± S.D.

Compared with the control group, the positive expressions of NF-κB in colon tissues in the model group increased significantly (*P* < 0.05). Compared with the model group, the positive expressions of NF-κB in colon tissues decreased in each administration group, with statistically significant differences (*P* < 0.05) (**Figure 4** and **Supplementary Materials Table S5**). Compared with the control group, the levels of BASO of whole blood in the model group increased significantly (*P* < 0.05). Compared with the model group, the levels of BASO of whole blood decreased in each administration group, with statistically significant differences (*P* < 0.05). Compared with the control group, the levels of LYMPH of whole blood decreased significantly (*P* < 0.05). Compared with the model group, the levels of LYMPH of whole blood increased in each administration group, with statistically significant differences (*P* < 0.05). Remarkably, BA-BBR NPs is the superior efficacy by reducing the expressions of NF-κB in colon tissues and regulating the levels of BASO and LYMPH of whole blood (**Figure 5**).

### Intestinal Flora

**Figure 6:**
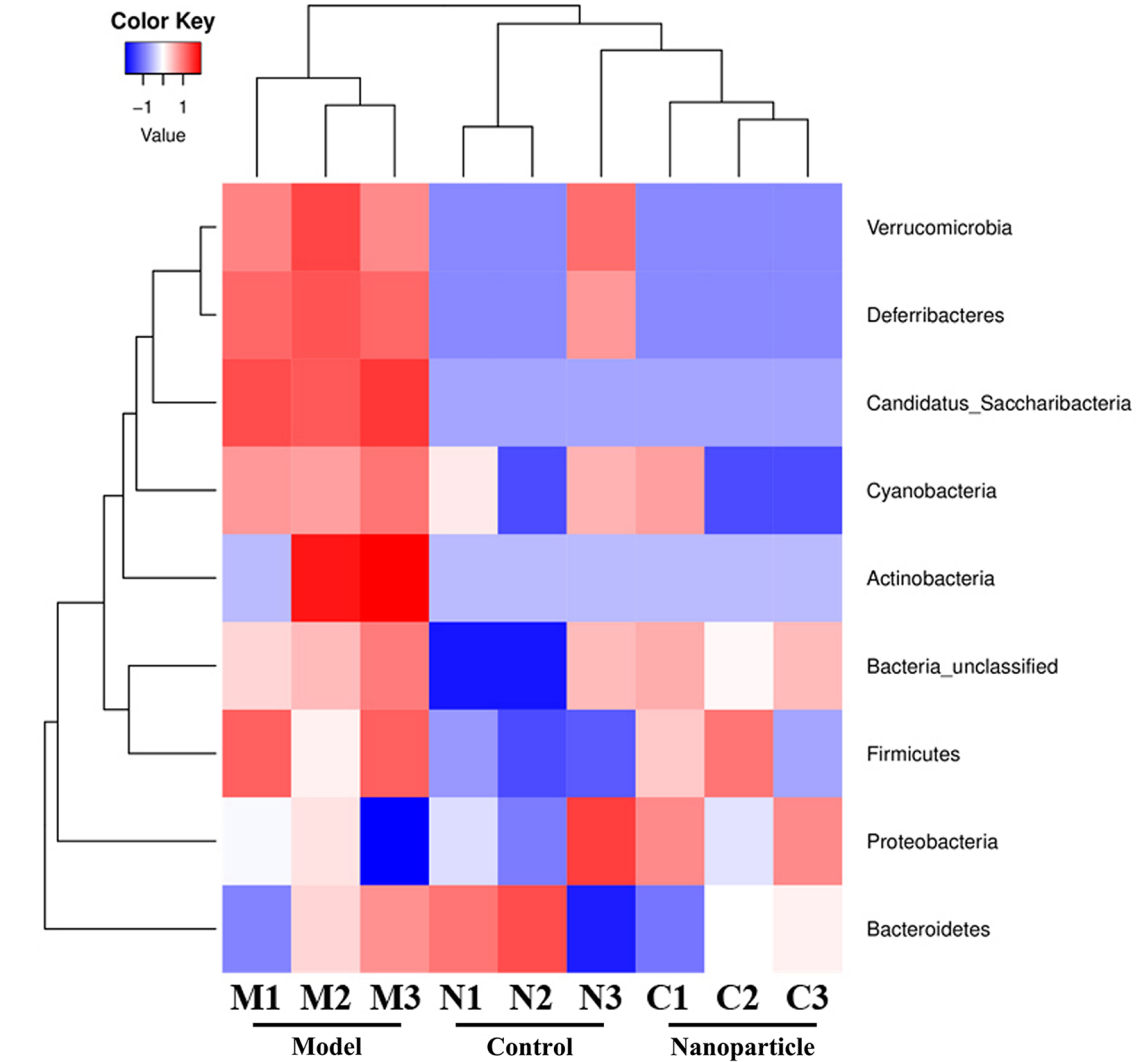
Heatmap analysis of 16s rRNA gene sequences of microbial samples isolated from the colons of control, model, and BA-BBR NPs group.

The overall structural changes of intestinal flora in response to instant BA-BBR NPs were determined by analysis of the 16S rRNA gene sequences of microbial samples isolated from the colon of control group, model group and BA-BBR NPs groups. UniFrac distance-based principal coordinate analysis (PCoA) revealed distinct clustering of intestinal microbe communities for each group. As shown in the PCoA plot, the relative abundances of Bacteroidia, Deferribacteres, Verrucomicrobia, Candidatus_Saccharibacteria, Cyanobacteria were all significantly increased in model group than in normal group, whereas BA-BBR NPs treatment reduced the relative abundances of these phylum than in model group. BA-BBR NPs treatment remarkably reduced the relative abundances of Verrucomicrobia compared with both the model group and control group (**Figure 6**, **Table 5**, and **Supplementary Materials Figure S1**).

**Table 5:** Partial results of sample difference analysis by 16s.

Phylum	p value	q value	Model	Control	Nanoparticle
p:Bacteria_unclassified	0.045	0.220	0.500 ± 0.001*	0.050 ± 0.100	0.040 ± 0.011^#^
p:Deferribacteres	0.012	0.220	0.800 ± 0.000*	0.205 ± 0.005	0.000 ± 0.213^#^
p:Verrucomicrobia	0.012	0.220	1.120 ± 0.000*	0.340 ± 0.012	0.000 ± 0.209^#^
p:Candidatus_Saccharibacteria	0.012	0.220	0.900 ± 0.000*	0.020 ± 0.004	0.015 ± 0.001^#^
p:Cyanobacteria	0.025	0.350	0.090 ± 0.000*	0.030 ± 0.021	0.020 ± 0.005^#^

ANOVA with the post hoc test was used to calculate the significance of the differences, * and ^#^ represents P < 0.05 compared with the control and model group, respectively. The results are expressed as the mean ± S.D.

## Discussion

Nowadays, regarding the multifactorial pathophysiology of IBS-D, appropriate treatment of IBS is still a challenge (Chey et al., 2015). However, TCM usually combine Scutellaria baicalensis Georgi and Coptis chinensis Franch rhizome to treat diarrhea (Chen et al., 2015). Inspired by the combination of TCM, we successfully gained self-assembled nanoparticles of BBR and BA, which were originated from the classic herb – drug combination. Here, we reported that BA-BBR NPs produced a synergistic effect on IBS-D mice, which might be related to BGPs, immune inflammation and intestinal flora from three important interrelated components of MGBA.

In our study, to ensure that the effects of BA-BBR NPs, BBR-BA mixture, BA, and BBR are comparable, we calculated the dose of these drugs according to previous studies about dose of BBR for IBS-D model animals basing on the dose - ranges of BBR for treating diarrhea in adults and the molecular weight ratio of BBR and BA in the nanoparticles (Chen et al., 2015). Remarkably, berberine hydrochloride tablet prepared by BBR was famous in clinic to treat diarrhea in China (Chen et al., 2015). Recently, more and more researchers showed that Phytogenic BBR could treat IBS-D through multiple ways (Hou et al., 2019; Qiong et al., 2019). In our study, we verified that BBR could improve diarrhea, visceral hypersensitivity, and depression, anxiety-like behaviors on IBS-D model mice. In addition, we found that BA could also improve these symptoms, whereas the effect is worse than BBR. Furthermore, BA-BBR NPs showed the best therapeutic effect on basic efficacy evaluation of IBS-D model mice, compared with BBR and BA, indicating that the synergistic efficacy of BBR had been gain after the formation of nanoparticles with BA. This result might provide an idea to explain the combination of Scutellaria baicalensis Georgi and Coptis chinensis Franch rhizome to treat diarrhea by TCM. Finally, BA-BBR NPs showed better therapeutic effect on IBS-D than BA/BBR mixture, suggesting that the synergistic effect of BBR and BA is achieved due to the formation of self-assemblies rather than the simple mixing, which is consistent with the results of our research *in vitro* (Zhang et al., 2016; Wang et al., 2017; Huang et al., 2020).

Having demonstrated the synergistic action, we next found that synergistic effect of BA-BBR NPs on IBS-D mice might be related to BGPs, immune inflammation and intestinal flora from three important interrelated components of MGBA. Studies have shown that 5-HT, VIP and CHAT plays the important role on inducing diarrhea, high visceral sensitivity, and depression on IBS-D of patients and rats (Tjong et al., 2011; Holzer and Farzi, 2014). Correspondingly, we examined these BGPs level in colon and serum in our study. The reason why we chose to detect BGPs in serum instead of that in brain was that the change trend of BGPs both in colon and serum consistent with the psychiatric symptoms of IBS-D patients (Holzer and Farzi, 2014; Brun et al., 2017), our preliminary experiments demonstrated that brain symptoms appeared such as depression, anxiety-like behaviors, and pain on IBS-D mice and the BGPs in serum was more suitable for dynamic observation than that in brain in a follow-up experiment. BGPs experimental results showed that BA-BBR NPs significantly reduced the levels of 5-HT, VIP, and CHAT in colon tissues or of serum compared with other drugs, indicating that the ameliorative synergistic action of BA-BBR NPs is attained by influencing BGPs. Recently, there are evidences for immune activation and low-grade mucosal inflammation, characterized by activating intestinal NF-κB signal pathway and abnormal amounts of immune cells, in the colon and ileum of IBS patients (Juan et al., 2014). Immune inflammation results showed that BA-BBR NPs significantly lowered the expressions of NF-κB in colon tissues and changed the levels of BASO and LYMPH in whole blood, compared with other drugs, suggesting that the synergistic effect of BA-BBR NPs could be attributed to the immune inflammation. Finally, our study preliminarily found that BA-BBR NPs could altered intestinal flora of Bacteroidia, Deferribacteres, Verrucomicrobia, Candidatus_Saccharibacteria, and Cyanobacteria; however, the relationship between these bacteria and IBS-D needed further study.

Therefore, BGPs, immune inflammation and intestinal flora from three important interrelated components of MGBA are participated in synergistic effect of BA-BBR NPs on IBS-D mice. However, the mechanism how these three aspects interact with each other under the action of BA-BBR NPs remains to be further explored, and thus might provide a medicinal plant-derived natural and synergistic efficient nanomaterial for IBS-D therapeutics.

## Conclusions

**Figure 7:**
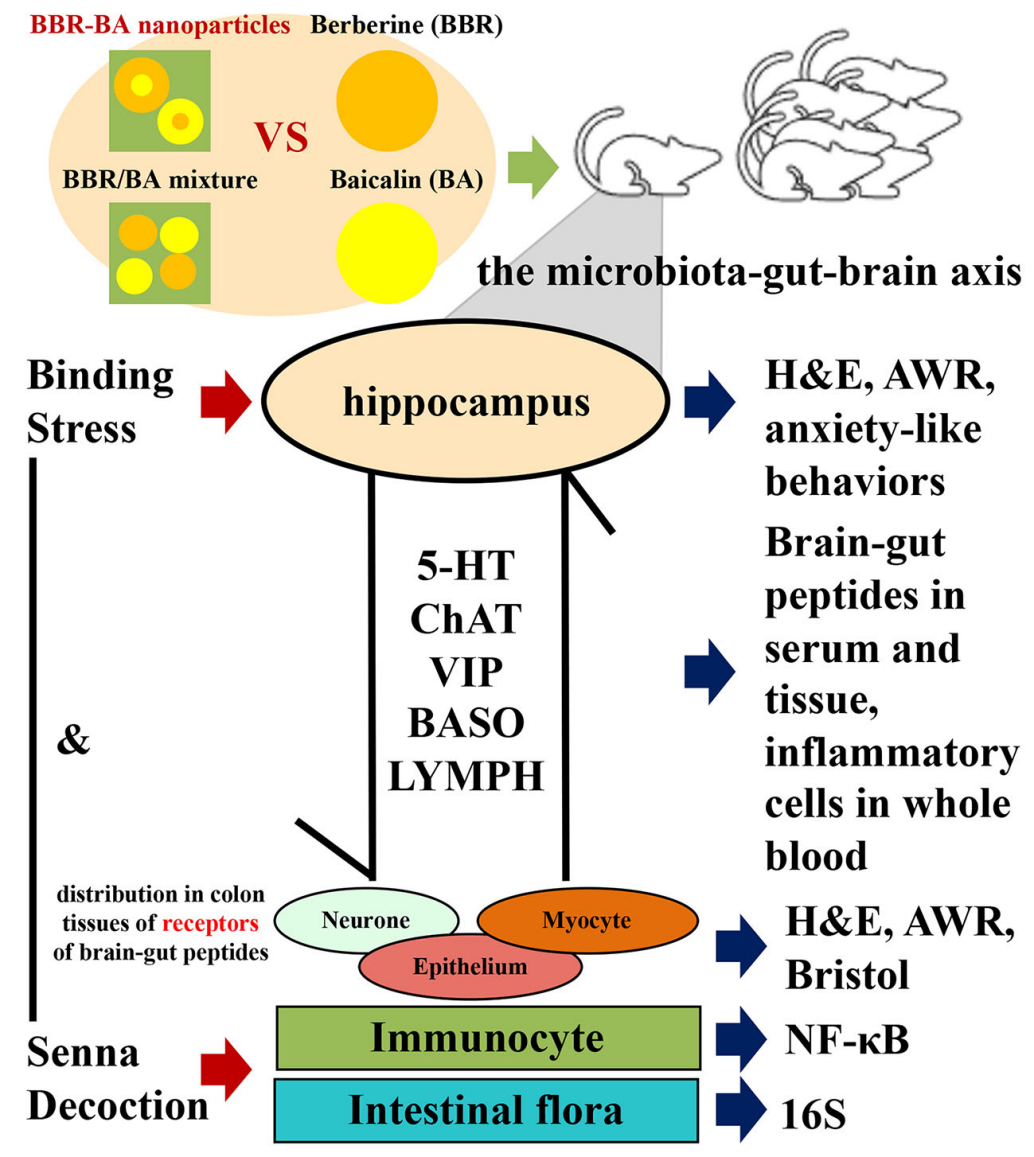
Diagram of this experiment.

In conclusion, BA-BBR NPs produced a synergistic effect on IBS-D mice, which is achieved due to the formation of self-assemblies rather than the simple mixing. Furthermore, we found that synergistic effect of BA-BBR NPs on IBS-D mice might be related to BGPs, immune inflammation, and intestinal flora from three important interrelated components of MGBA. This study will provide a novel idea for the interpretation of TCM compatibility theory and provide the basis for BA-BBR NPs as a medicinal plant-derived natural and synergistic efficient nanomaterial for IBS-D therapeutics from the enlightenment of TCM combination (**Figure 7**).

## Data Availability Statement

The raw data supporting the conclusions of this article will be made available by the authors, without undue reservation, to any qualified researcher.

## Ethics Statement

The animal study was reviewed and approved by: The protocol was approved by the Beijing University of Chinese Medicine Medical and Experimental Animal Ethics Committee. Animal ethics review number was BUCM-4-2019040401-2003.

## Author Contributions

PW, HL, and CW conceived, designed and provided detailed guidance on the implementation of this experiment. PW, HX, FC, and BX directed the conduct of the experiments. LL and HCu performed the all experiments. TL, JQ, HCh, FG, YM, and RH participated in the experiment. XT and SL conducted data analysis and statistics. LL and HC wrote, revised, and improved the paper. All authors contributed to the article and approved the submitted version.

## Funding

This research was supported by National Natural Science Foundation of China (No. 8157150997), Beijing Nova program (No. Z201100006820026), the Beijing Municipal Natural Science Foundation (No. 7202116), National Natural Science Foundation of China (No. 81603256), project of China Association of Chinese Medicine (CACM-2018-QNRC2-B08), the Fundamental Research Funds for the Central Universities (2020-JYB-ZDGG-044, BUCM-2019-JCRC002, and 2019-JYB-TD005, China), Beijing Key Laboratory for Basic and Development Research on Chinese Medicine (Beijing, 100102).

## Conflict of Interest

The authors declare that the research was conducted in the absence of any commercial or financial relationships that could be construed as a potential conflict of interest.
