# Supplementary material for: Synergistic Effect of Berberine-Based Chinese Medicine Assembled Nanostructures on Diarrhea-Predominant Irritable Bowel Syndrome In Vivo
Source: Front Pharmacol. 2020 Aug 31;11:1210. doi: 10.3389/fphar.2020.01210 (PMC7490548; doi:10.3389/fphar.2020.01210)

***Supplementary Materials for***

**Synergistic effect of berberine-based Chinese medicine assembled nanostructures on diarrhea-predominant irritable bowel syndrome *in vivo***

**Lei Li^1,2#^, Herong Cui^2#^, Tong Li^2^, Jinchai Qi^2^, Hongshan Chen^2^, Feng Gao^2^,** **Xuehao Tian^2^**,**Yunnong Mu^4^, Rui He^4^, Siyuan Lv^1^, Fuhao Chu^5^, Bing Xu^2^, Penglong Wang^2*^, Haimin Lei^2*^,Hongri Xu^3*^, Chengxiang Wang^1*^**

^1^Respiratory Department, Beijing University of Chinese Medicine (BUCM) Third Affiliated Hospital, Beijing, China;

^2^School of Chinese Pharmacy, Beijing University of Chinese Medicine, Beijing China;

^3^Emergency Department, BUCM Third Affiliated Hospital, Beijing, China;

^4^School of Acupuncture-Moxibustion and Tuina, Beijing University of Chinese Medicine, Beijing China;

^5^School of Traditional Chinese Medicine, Beijing University of Chinese Medicine, Beijing China

List of supplementary materials

| No. | Content | Figure/Table |
| --- | --- | --- |
| 1 | 5-HT in colon tissue | Table S1 |
| 2 | VIP in colon tissue | Table S2 |
| 3 | 5-HT of serum | Table S3 |
| 4 | ChAT of serum | Table S4 |
| 5 | Relative expression of NF-κB in colon tissue | Table S5 |
| 6 | ROC curve of 16s | Figure S1 |

1. 5-HT in colon tissue.

| **Table S1** 5-HT in colon tissue (ng/mL) | | |
| --- | --- | --- |
| **group** | **mean** | **S.D.** |
| Normal | 92.38 | 16.43 |
| Model | 193.08 | 22.66 |
| Complex | 56.83 | 17.71 |
| Mixture | 154.27 | 2.33 |
| Baicalin | 112.53 | 8.11 |
| Berberine | 72.20 | 14.72 |

2. VIP in colon tissue.

| **Table S2** VIP in colon tissue (ng/L) | | |
| --- | --- | --- |
| **group** | **mean** | **S.D.** |
| Normal | 128.75 | 23.36 |
| Model | 358.05 | 110.31 |
| Complex | 71.18 | 2.48 |
| Mixture | 141.78 | 17.73 |
| Baicalin | 314.73 | 95.70 |
| Berberine | 113.60 | 9.53 |

3. 5-HT of serum.

| **Table S3** 5-HT of serum (ng/mL) | | |
| --- | --- | --- |
| **group** | **mean** | **S.D.** |
| Normal | 58.81 | 22.55 |
| Model | 117.56 | 13.21 |
| Complex | 41.71 | 4.06 |
| Mixture | 76.02 | 11.64 |
| Baicalin | 104.21 | 31.19 |
| Berberine | 67.71 | 5.51 |

4. ChAT of serum.

| **Table S4** ChAT of serum (U/L) | | |
| --- | --- | --- |
| **group** | **mean** | **S.D.** |
| Normal | 258.82 | 87.08 |
| Model | 371.62 | 5.92 |
| Complex | 196.11 | 12.41 |
| Mixture | 234.18 | 3.20 |
| Baicalin | 147.59 | 31.96 |
| Berberine | 264.74 | 19.93 |

5. Relative expression of NF-κB in colon tissue.

| **Table S5** Relative expression of NF-κB in colon tissue | | |
| --- | --- | --- |
| **group** | **mean** | **S.D.** |
| Normal | 24.41 | 1.06 |
| Model | 33.65 | 5.75 |
| Complex | 23.32 | 4.38 |
| Mixture | 32.64 | 6.07 |
| Baicalin | 32.37 | 9.87 |
| Berberine | 37.58 | 2.34 |

6. ROC curve of 16s.


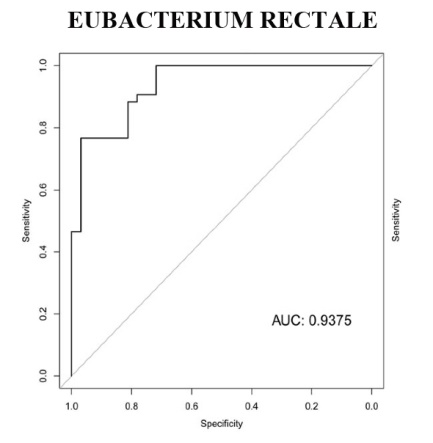

Supplement: Supplementary file 1 [file DataSheet_1.docx]
